# AppleLeafNet: a lightweight and efficient deep learning framework for diagnosing apple leaf diseases

**DOI:** 10.3389/fpls.2024.1502314

**Published:** 2024-11-27

**Authors:** Muhammad Umair Ali, Majdi Khalid, Majed Farrash, Hassan Fareed M. Lahza, Amad Zafar, Seong-Han Kim

**Affiliations:** ^1^ Department of Artificial Intelligence and Robotics, Sejong University, Seoul, Republic of Korea; ^2^ Department of Computer Science and Artificial Intelligence, College of Computing, Umm Al-Qura University, Makkah, Saudi Arabia; ^3^ Department of Cybersecurity, College of Computing, Umm Al-Qura University, Makkah, Saudi Arabia

**Keywords:** deep learning, apple leaf condition identification, apple leaf disease detection, lightweight model, crop monitoring

## Abstract

Accurately identifying apple diseases is essential to control their spread and support the industry. Timely and precise detection is crucial for managing the spread of diseases, thereby improving the production and quality of apples. However, the development of algorithms for analyzing complex leaf images remains a significant challenge. Therefore, in this study, a lightweight deep learning model is designed from scratch to identify the apple leaf condition. The developed framework comprises two stages. First, the designed 37-layer model was employed to assess the condition of apple leaves (healthy or diseased). Second, transfer learning was used for further subclassification of the disease class (e.g., rust, complex, scab, and frogeye leaf spots). The trained lightweight model was reused because the model trained with correlated images facilitated transfer learning for further classification of the disease class. A dataset available online was used to validate the proposed two-stage framework, resulting in a classification rate of 98.25% for apple leaf condition identification and an accuracy of 98.60% for apple leaf disease diagnosis. Furthermore, the results confirm that the proposed model is lightweight and involves relatively fewer learnable parameters in comparison with other pre-trained deep learning models.

## 1 Introduction

Ensuring constant and steady agricultural production is crucial for satisfying the demands of the growing global population. Agricultural production and quality vary globally owing to various factors, such as climatic changes, naturally available resources, geographical position, and the presence of infections and diseases (Mahato et al., 2022). Additionally, plant diseases contribute to insufficient human food supply and may severely impact natural ecosystems (Singh et al., 2023; Lahlali et al., 2024). Although technological advancements have mitigated the catastrophic effects of plant diseases, this remains a significant issue.

With a history of 2,000 years of human cultivation, apples are one of the most popular and extensively cultivated fruits globally (Cheng and Li, 2023). Apples are rich in vitamins and minerals, which provide a high nutritional value that is essential for a healthy diet (Korban, 2023; Tsoupras et al., 2023) (Larsson et al., 2013). have reported that the adequate consumption of apples may reduce the risk of stroke. However, apple production faces various challenges because of diseases that can significantly affect both yield and quality. Apple trees are vulnerable to a multitude of diseases that significantly compromise their quality and yield. These include fungal infections, viruses, nematodes, and bacteria, which can substantially reduce the nutritional and therapeutic value of apples. At present, disease identification significantly relies on human vision, requiring the expertise of local agriculturalists (Dutot et al., 2013). In addition to being cumbersome, visual inspection by farmers is susceptible to errors because of subjective perceptions and visual fatigue, rendering it challenging to achieve high precision during disease identification. This can cause significant losses in apple production and quality. Diseases such as rust, complexes, scabs, and frogeye leaf spots hinder apple production, inducing significant setbacks in the agricultural sector. Rust reduces fruit size and places trees at risk of harm during winters. Cedar-apple rust affects leaves and fruits, whereas frogeye leaf spots, caused by a fungal pathogen, lead to fruit infections. The scab, caused by Venturia inaequalis, is particularly damaging; it begins as yellow spots on leaves and deforms the fruit subsequently (Hirst, 1997). Therefore, the timely and accurate diagnosis and treatment of apple diseases are essential to ensure a productive and healthy harvest. Over the last few years, advancements in machine learning and deep learning technologies have significantly enhanced the detection of leaf diseases (Kamilaris and Prenafeta-Boldú, 2018; Pardede et al., 2018), facilitating efficient real-time disease detection.

Recognizing plant diseases is fundamentally an image-processing problem that involves accurate capturing of disease features, comparing them with other disease types, and classifying them. Conventional machine learning approaches employ image processing methods and classifiers, wherein RGB values and disease spot textures are extracted using grayscale values. The commonly used classifiers include Naive Bayes, support vector machines, and k-means clustering (Mokhtar et al., 2015; Ma et al., 2018; Deng et al., 2019; Tian et al., 2019). Conventional machine learning methods can achieve reasonable detection rates for diseases with specific characteristics (Singh et al., 2016). However, these approaches are constrained by their inadequacy in identifying nonlinear data and the challenges associated with feature extraction, resulting in inadequate generalizability.

By contrast, deep learning methods have reported promising results for plant disease detection (Fuentes et al., 2017; Liu et al., 2018). Deep learning models have demonstrated significantly accurate results in plant disease detection compared with conventional learning models. The automatic extraction of local features from neighboring pixels in deep learning models enables them to demonstrate high disease detection rates (Jiang et al., 2019). proposed a VGG-INCEP model for multiclassification problems. They used an apple leaf disease dataset to detect five different apple diseases and achieved an accuracy of 97.14%. In another study (Li and Rai, 2020), used pre-trained models, such as ResNet-18 and ResNet-34, for apple disease detection; the models achieved high accuracies of 99% and 97%, respectively, owing to their complex architectures. Although these models exhibit exceptional results, they are impractical for real-time use. In a recent study (Yao et al., 2024), introduced a multi-prediction model to identify plant diseases. The framework included a convolutional neural network (CNN) evaluated using a plant village, plant leaves, and PlantDoc datasets; their model exhibits a high detection rate of 96.51%. In another recent study (Andrushia et al., 2024), proposed a capsule network for classifying Vitis vinifera leaves, achieving an accuracy of 98.7%. Recently, transformer-based architectures have revolutionized the field of agricultural image analysis, enabling accurate and efficient detection of apple leaf diseases. Several studies have demonstrated the effectiveness of transformer-based architectures in capturing contextual relationships and long-range dependencies, which are crucial for identifying subtle disease symptoms (Lv and Su, 2024; Si et al., 2024; Ullah et al., 2024). Despite their advantages, deep learning models, including transformer-based models, face significant challenges such as complex architectures, leading to increased computational requirements and substantial training time to achieve optimal results.

To address the aforementioned challenges, we developed a novel deep learning architecture and framework, referred to as AppleLeafNet, for apple disease identification and detection. In the first step, a deep learning classification architecture was designed from scratch to identify the condition of the apple leaf (healthy or diseased). After identifying the condition of the leaf, the same deep learning model was reused considering its frozen weight (transfer learning concept) for subclassifying the diseased leaf into rust, complex, scab, and frogeye leaf spot. The use of frozen weights on correlated images facilitated the subclassification process. A dataset available online was used to validate the proposed deep learning model and framework.

## 2 Materials and methods

This section presents the details of the materials used and the methods employed in this study, including the proposed model and framework. The data augmentation process, implemented to address class imbalance issues in the dataset used, is also described.

### 2.1 Proposed framework for apple leaf disease detection

**Figure 1:**
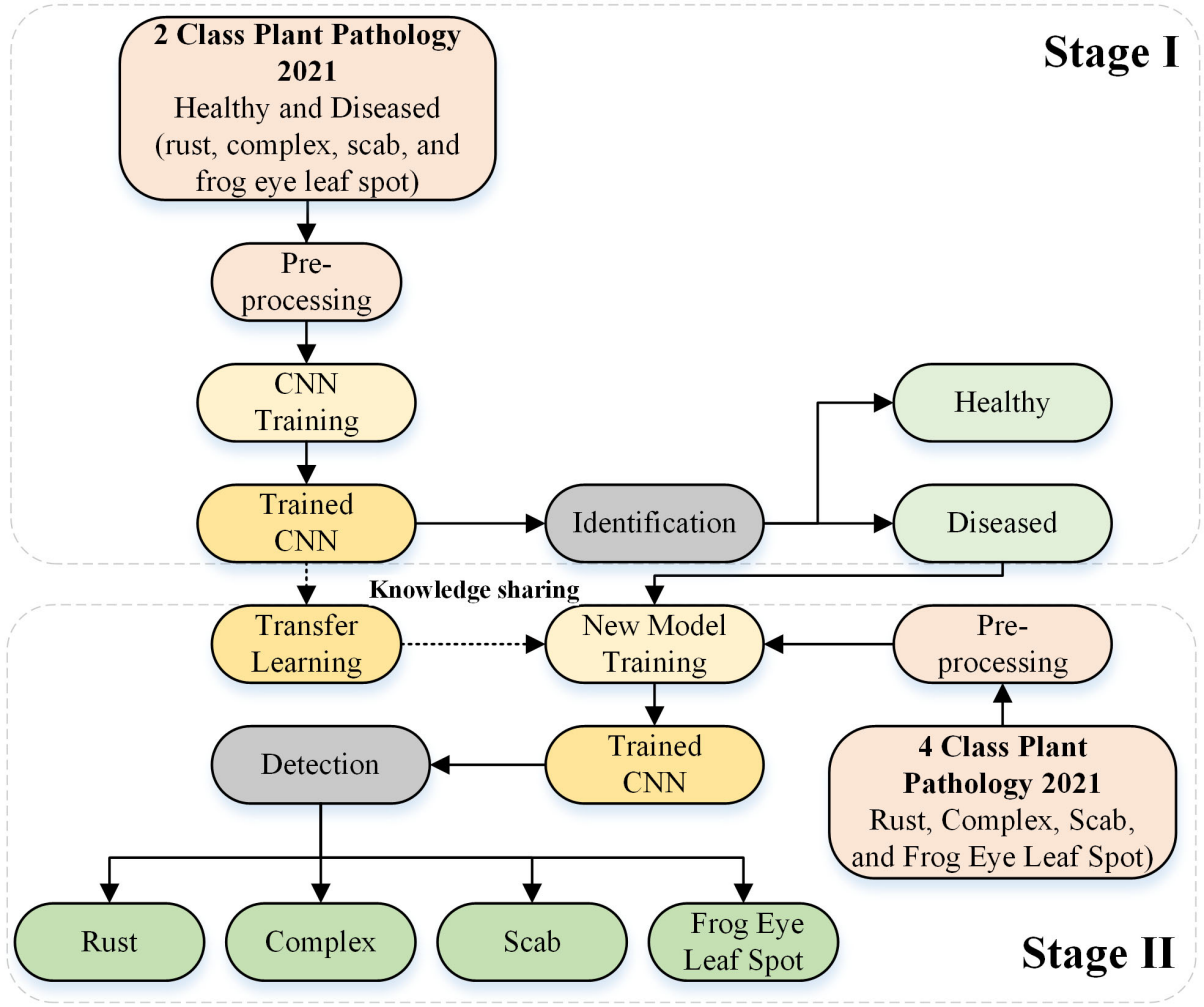
Framework used for apple leaf disease identification and detection based on the designed deep learning model.

We propose a deep-learning-based framework for identifying and detecting the types of actual diseases that affect apple leaves. The proposed approach was divided into two stages. In Stage I, the deep learning architecture was designed from scratch to identify the condition of the apple leaf (healthy or diseased). In Stage II, the diseased leaves were further categorized into rust, complex, scab, and frogeye leaf spots by reusing the trained Stage I model. **Figure 1** depicts the complete framework of the proposed approach. The details of the deep learning model are provided in the subsequent section.

### 2.2 Deep learning model designed from scratch

**Figure 2:**
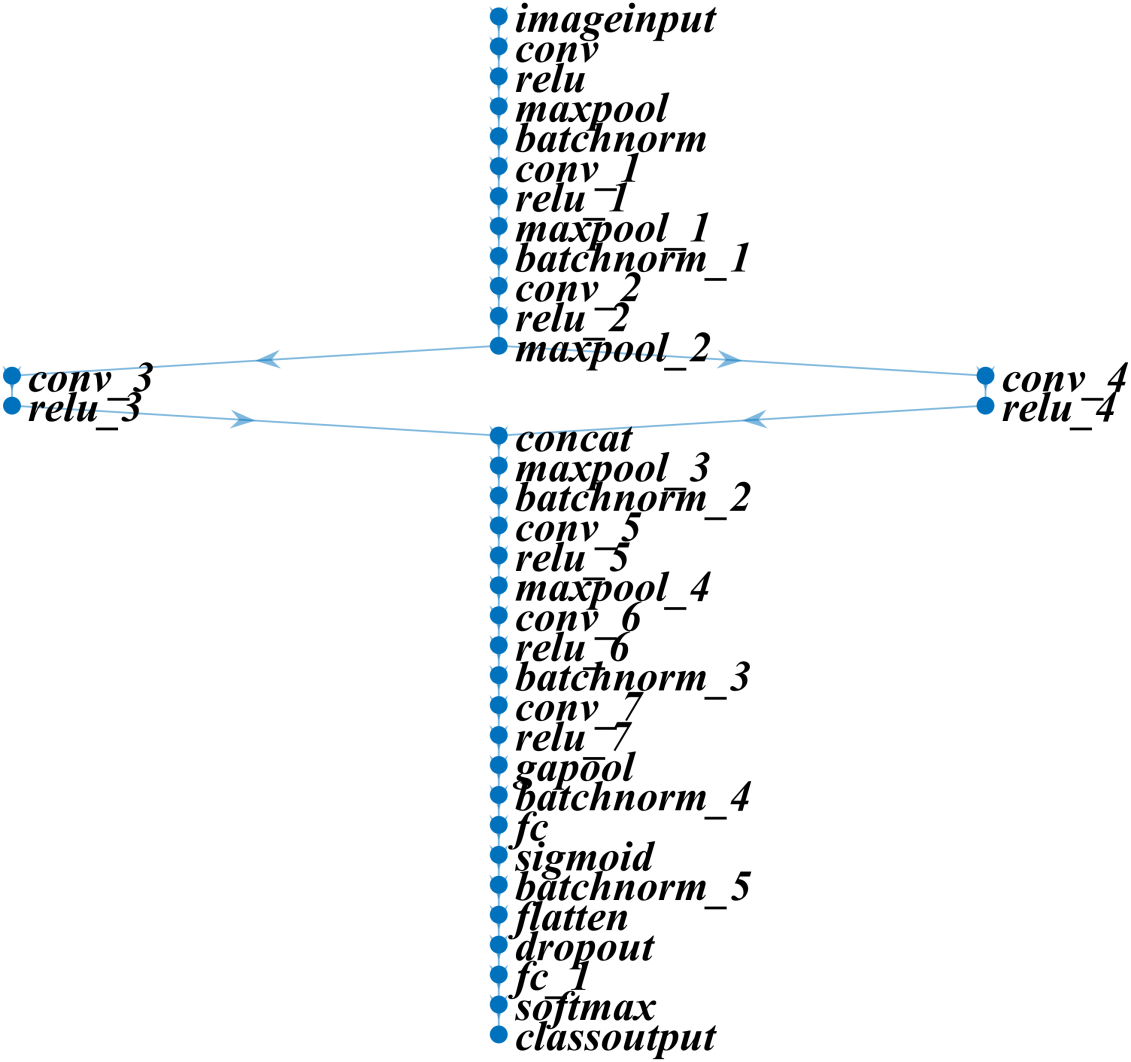
Architecture of the developed deep learning model for apple leaf disease identification.

In this study, we developed a deep learning model for apple leaf disease identification. The proposed model was designed to outperform other state-of-the-art models by using the fewest layers possible for a specific dataset. The proposed model was built using 37 layers, which included the input, convolutional, rectified linear unit (ReLU), pooling, batch normalization, concatenation, fully connected, dropout, softmax, and classification layers. **Figure 2** and **Table 1** present the specifications and features of the proposed deep learning model.

**Table 1:** Detailed information on the developed deep learning model for identifying the conditions of apple leaves.

Layer No.	Name	Type	Activations	No. of Learnable Parameters
1	‘imageinput’	Image Input	[227,227,3,1]	0
2	‘conv’	2-D Convolution	[227,227,32,1]	896
3	‘relu’	ReLU	[227,227,32,1]	0
4	‘maxpool’	2-D Max Pooling	[113,113,32,1]	0
5	‘batchnorm’	Batch Normalization	[113,113,32,1]	64
6	‘conv_1’	2-D Convolution	[113,113,32,1]	9,248
7	‘relu_1’	ReLU	[113,113,32,1]	0
8	‘maxpool_1’	2-D Max Pooling	[56,56,32,1]	0
9	‘batchnorm_1’	Batch Normalization	[56,56,32,1]	64
10	‘conv_2’	2-D Convolution	[56,56,64,1]	18,496
11	‘relu_2’	ReLU	[56,56,64,1]	0
12	‘maxpool_2’	2-D Max Pooling	[27,27,64,1]	0
13	‘conv_3’	2-D Convolution	[27,27,64,1]	36,928
14	‘relu_3’	ReLU	[27,27,64,1]	0
15	‘conv_4’	2-D Convolution	[27,27,64,1]	36,928
16	‘relu_4’	ReLU	[27,27,64,1]	0
17	‘concat’	Concatenation	[27,54,64,1]	0
18	‘maxpool_3’	2-D Max Pooling	[13,26,64,1]	0
19	‘batchnorm_2’	Batch Normalization	[13,26,64,1]	128
20	‘conv_5’	2-D Convolution	[13,26,128,1]	73,856
21	‘relu_5’	ReLU	[13,26,128,1]	0
22	‘maxpool_4’	2-D Max Pooling	[6,12,128,1]	0
23	‘conv_6’	2-D Convolution	[6,12,256,1]	295,168
24	‘relu_6’	ReLU	[6,12,256,1]	0
25	‘batchnorm_3’	Batch Normalization	[6,12,256,1]	512
26	‘conv_7’	2-D Convolution	[6,12,256,1]	590,080
27	‘relu_7’	ReLU	[6,12,256,1]	0
28	‘gapool’	2-D Global Average Pooling	[1,1,256,1]	0
29	‘batchnorm_4’	Batch Normalization	[1,1,256,1]	512
30	‘fc’	Fully Connected	[1,1,1024,1]	263,168
31	‘sigmoid’	Sigmoid	[1,1,1024,1]	0
32	‘batchnorm_5’	Batch Normalization	[1,1,1024,1]	2,048
33	‘flatten’	Flatten	[1024,1]	0
34	‘dropout’	Dropout	[1024,1]	0
35	‘fc_1’	Fully Connected	[2,1]	2,050
36	‘softmax’	Softmax	[2,1]	0
37	‘classoutput’	Classification Output	[2,1]	0

#### 2.2.1 Input layer

The model uses the input image to extract the features from the subsequent layers. The developed structure was designed to process input images with dimensions of 227 × 227 × 3. Each image in the dataset was adjusted to fit these dimensions; this relates only to the input image dimensions and contains no learning parameters, as listed in **Table 1**.

#### 2.2.2 Convolutional layer

The layers of the model learn weight matrices for filters and kernels, with the number and size of filters determining the adjustable parameters. For instance, in a layer with dimensions (x, y, d) and a filter size k with dimensions (a, b), the parameters of the convolutional layer are (a * b * d) + 1) * k), where 1 is included for the bias in each filter. In this study, the presented network included eight convolutional layers with a filter size of 3 × 3, resulting in 1,061,600 learnable parameters across the layers.

#### 2.2.3 Pooling layer

This layer reduces the number of input parameters to decrease computation costs and enhance efficiency. The developed model includes max- and average-pooling layers; the max-pooling layer selects the most prominent features, whereas the average-pooling layer computes the average value based on the feature map using stride and padding settings. This layer lowers the input dimensions but does not contain learnable parameters.

#### 2.2.4 Dropout layer

This layer randomly deactivates neurons during training, prevents overfitting, and enhances the model generalization.

#### 2.2.5 Fully connected layer

This layer establishes dense connections, thereby extracting high-level characteristics to develop the classification model.

#### 2.2.6 Softmax

Softmax uses an activation function to transform logarithms into class probabilities, ensuring that the sum of the probabilities for all classes is equal to one.

#### 2.2.7 Classification output

This layer employs cross-entropy loss for model training, explicitly using the “crossentropyex” of MATLAB. The gap between the actual and predicted class probabilities can be quantified using cross-entropy.

The network developed from scratch for disease identification contained only 1,330,146 learnable parameters, as listed in **Table 1**.

### 2.3 Transfer learning

Transfer learning is a machine learning method that uses existing trained models to accelerate learning for another task. Essentially, the network created for one task is reused as the initial point of another network for a different task. This approach is particularly valuable when limited datasets are available. The fundamental idea of this approach is to utilize the characteristics acquired from tasks with ample data to enhance the efficiency of tasks with limited data. This is based on the understanding that tasks involve common elements that can be reused to enhance efficiency.

In Stage II of the framework, transfer learning was employed based on the deep learning model developed in Stage I by incorporating the frozen weights of the apple disease identification model (trained for Stage I). The network was retrained by substituting the final three layers for actual disease detection (e.g., rust, complex, scab, and frogeye leaf spots), as indicated in **Figure 1**.

### 2.4 Dataset and preprocessing

**Figure 3:**
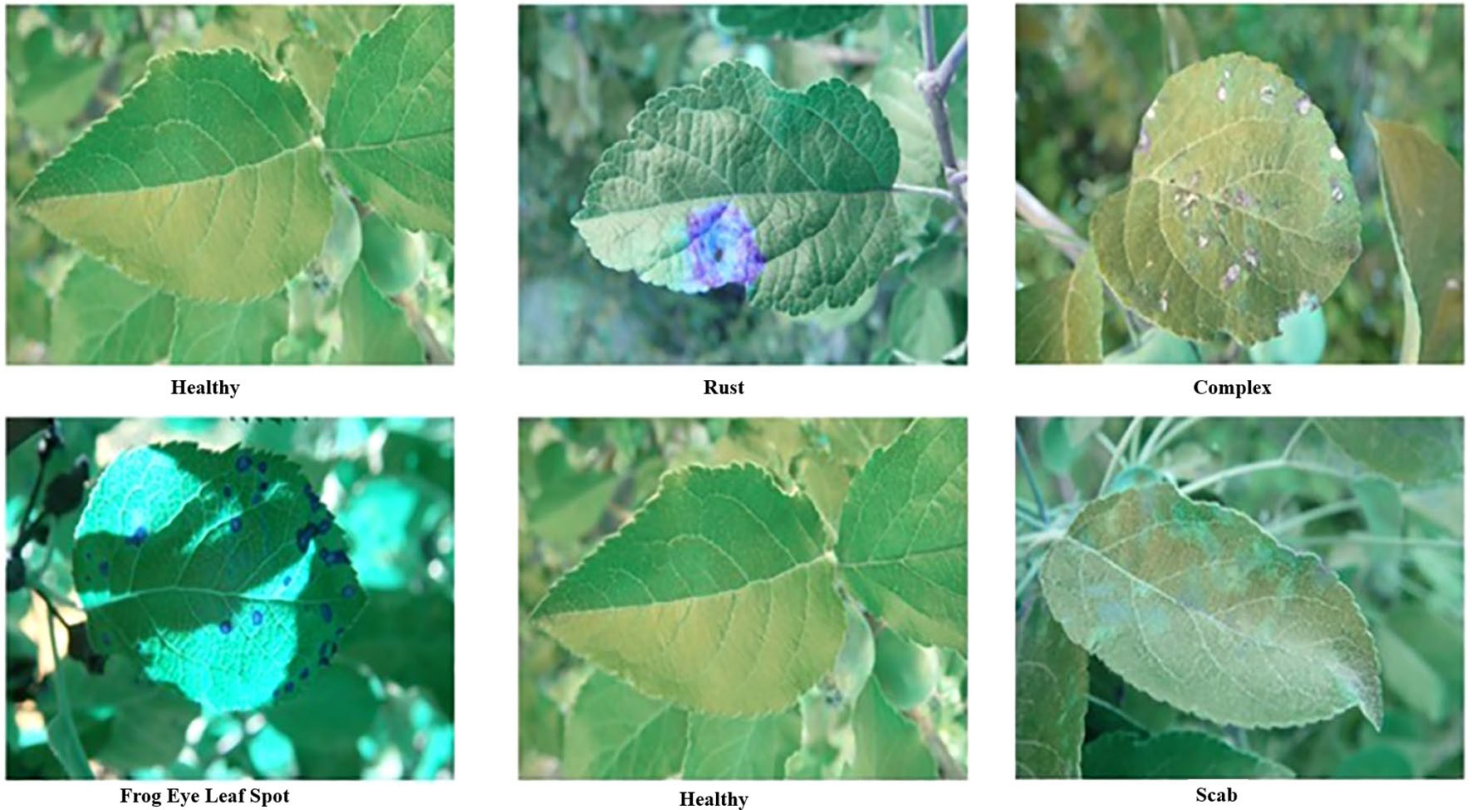
Sample of the plant pathology 2021 - FGVC8 dataset used for various conditions of leaves.

The dataset used in this study is publicly available at Kaggle “Plant Pathology 2021 - FGVC8” (https://www.kaggle.com/competitions/plant-pathology-2021-fgvc8/data, accessed on August 20, 2024). The dataset contains 18,632 images captured using a Canon Rebel T5i DSLR (Canon Inc., Japan) and is mobile at different angles, illumination, noise, and non-homogeneous backgrounds, depicting various disease levels. According to the input of the developed model, all images were uniformly cropped to 227 × 227 pixels. A zero-center approach was used for normalization during preprocessing. We selected five leaf spot categories, namely, healthy, rust, complex, scab, and frogeye leaf spots, based on their sufficient image representation (**Figure 3**).

Insufficient data is a major challenge in implementing deep learning models. Increasing the number of images in the dataset can help models learn robust features and reduce the risk of overfitting. The dataset used in this study was imbalanced, with the scab class containing the maximum images and the complex class comprising the least number of images. We addressed this imbalance by using techniques such as flipping, random rotation, contrast adjustment, brightness modification, translation, and zoom to enhance the dataset. These augmentations increased the size of the dataset and improved image quality. The dataset was divided into two groups, as summarized in **Table 2**.

**Table 2:** Details of the apple leaf dataset.

Category	Subcategory	Original Images	Augmented Images for Stage I	Augmented Images for Stage II
Healthy	Healthy	4624	6000	
Diseased	Rust	1860	6000	6000
	Complex	1602		6000
	Scab	4826		6000
	Frogeye Leaf Spots	3181		6000

## 3 Results

MATLAB 2023a was used for all simulations and analyses on a personal computer with the following specifications: Core i7, 12th Generation, 32 GB RAM, NVIDIA GeForce RTX 3050, 1 TB SSD, and 64-bit Windows 11 operating system. The dataset was randomly divided into 80/20 ratios for model training and testing. The images used for the model testing were not part of the training set. The initial parameters included 100 epochs, a momentum of 0.9, a mini-batch size of 32, and a learning rate of 0.001. The stochastic gradient descent with momentum (SGDM) solver was used for training and testing. The following metrics were used to evaluate the performance of the various models:


$$\text{Precision}=\frac{\text{True positives}}{\text{True positives}+\text{False positives}}$$

$$\text{Recall}=\frac{\text{True positives}}{\text{True positives}+\text{False negatives}}$$

$$\text{Specificity}=\frac{\text{True negatives}}{\text{True negatives}+\text{False positives}}$$

$$\text{F}1-\text{score}=2\times \frac{\text{Precision}\times \text{Recall}}{\text{Precision}+\text{Recall}}$$

$$\text{Accuracy}=\frac{\text{Total no}.\text{ of correctly classified observation}}{\text{Total no}.\text{ of observation}}$$

First, the original dataset (without augmentation) was used to verify the performance of the proposed model on a five-class apple leaf classification problem. Initially, an ablation study was performed to select the layers for the lightweight deep learning model. **Table 3** lists the results of the ablation study. The 37-layer model was selected as the proposed lightweight deep learning model because it yielded higher accuracy. Other state-of-the-art models, ranging from simple to complex architectures, such as ResNet-50, GoogLeNet, Inception-v3, EfficientNet-b0, MobileNet-v2, and DenseNet-201, were also trained to compare their performances. **Table 4** presents the results of the comparative analysis.

**Table 3:** Results of the ablation study performed for the selection of layers.

Parameters	Developed lightweight deep learning models				
	36-layer (no parallel branch)	33-Layer (1 parallel branch)	37-Layer (1 parallel branch)	41-Layer (1 parallel branch)	42-Layer (2 parallel branch)
Training Loss	1.39 × 10^-04^	4.42 × 10^-02^	6.30 × 10^-03^	1.17 ×10^-03^	7.72 × 10^-02^
Training Accuracy (%)	100	100	100	100	100
Validation Loss	0.51710	0.51105	0.4818	0.58545	0.51386
Validation Accuracy (%)	90.15	89.12	91.02	89.83	89.84
Training Time	65 min 30 s	59 min 3 s	61 min 38 s	63 min 38 s	76 min 57 s

**Table 4:** Comparison of various models for apple leaf disease detection.

Network	True Class	Predicted Class					Precision	Recall	Specificity	F1-score	Accuracy (%)	Training Time	Learnable (M)
		Complex	Frogels^*^	Healthy	Rust	Scab							
GoogLeNet	Complex	231	41	1	37	10	0.82	0.72	0.98	0.77	93.1	142 min 11 s	5.9
	Frogels^*^	20	604	4	2	6	0.92	0.95	0.98	0.93			
	Healthy	1	2	897	1	24	0.96	0.97	0.98	0.97			
	Rust	15	3	1	353	0	0.90	0.95	0.99	0.92			
	Scab	16	7	30	1	911	0.96	0.94	0.98	0.95			
ResNet-50	Complex	238	34	4	22	22	0.83	0.74	0.98	0.78	93.57	554 min 1 s	23.5
	Frogels^*^	28	600	4	1	3	0.92	0.94	0.98	0.93			
	Healthy	0	2	908	1	14	0.95	0.98	0.98	0.97			
	Rust	13	4	2	352	1	0.94	0.95	0.99	0.94			
	Scab	9	9	34	0	913	0.96	0.95	0.98	0.95			
Inception-v3	Complex	212	54	0	34	20	0.84	0.66	0.99	0.74	92.64	490 min 1 s	21.8
	Frogels^*^	21	597	4	6	8	0.90	0.94	0.97	0.92			
	Healthy	1	3	900	0	21	0.96	0.97	0.98	0.97			
	Rust	11	3	2	353	3	0.90	0.95	0.99	0.92			
	Scab	8	9	29	0	919	0.95	0.95	0.98	0.95			
EfficientNet-b0	Complex	252	37	0	10	8	0.80	0.82	0.98	0.81	93.8	653 min 21 s	4
	Frogels^*^	25	585	0	3	8	0.91	0.94	0.98	0.93			
	Healthy	1	5	907	0	29	0.98	0.96	0.99	0.97			
	Rust	20	1	0	356	1	0.96	0.94	0.99	0.94			
	Scab	19	8	18	3	919	0.95	0.95	0.98	0.95			
MobileNet-v2	Complex	246	20	0	16	9	0.77	0.86	0.97	0.81	93.9	230 min 32 s	2.2
	Frogels^*^	32	606	3	5	8	0.95	0.93	0.98	0.94			
	Healthy	0	7	904	1	32	0.98	0.96	0.99	0.97			
	Rust	30	0	0	350	0	0.94	0.92	0.99	0.93			
	Scab	12	3	18	0	916	0.95	0.96	0.98	0.96			
DenseNet-201	Complex	239	15	0	17	8	0.75	0.86	0.97	0.80	94.0	2700 min 27 s	18.1
	Frogels^*^	26	613	2	10	6	0.96	0.93	0.99	0.95			
	Healthy	2	3	915	1	37	0.99	0.96	0.99	0.97			
	Rust	29	1	0	343	0	0.92	0.92	0.99	0.92			
	Scab	24	4	8	1	914	0.95	096	0.98	0.95			
Proposed	Complex	229	43	7	31	10	0.85	0.72	0.99	0.78	91.02	61 min 17 s	1.3
	Frogels^*^	19	586	11	3	17	0.91	0.92	0.98	0.91			
	Healthy	1	3	875	0	46	0.92	0.95	0.97	0.93			
	Rust	16	9	0	343	4	0.91	0.92	0.99	0.92			
	Scab	4	6	59	0	896	0.92	0.93	0.97	0.92			

Frogels^*^, Frogeye Leaf Spots.

**Figure 4:**
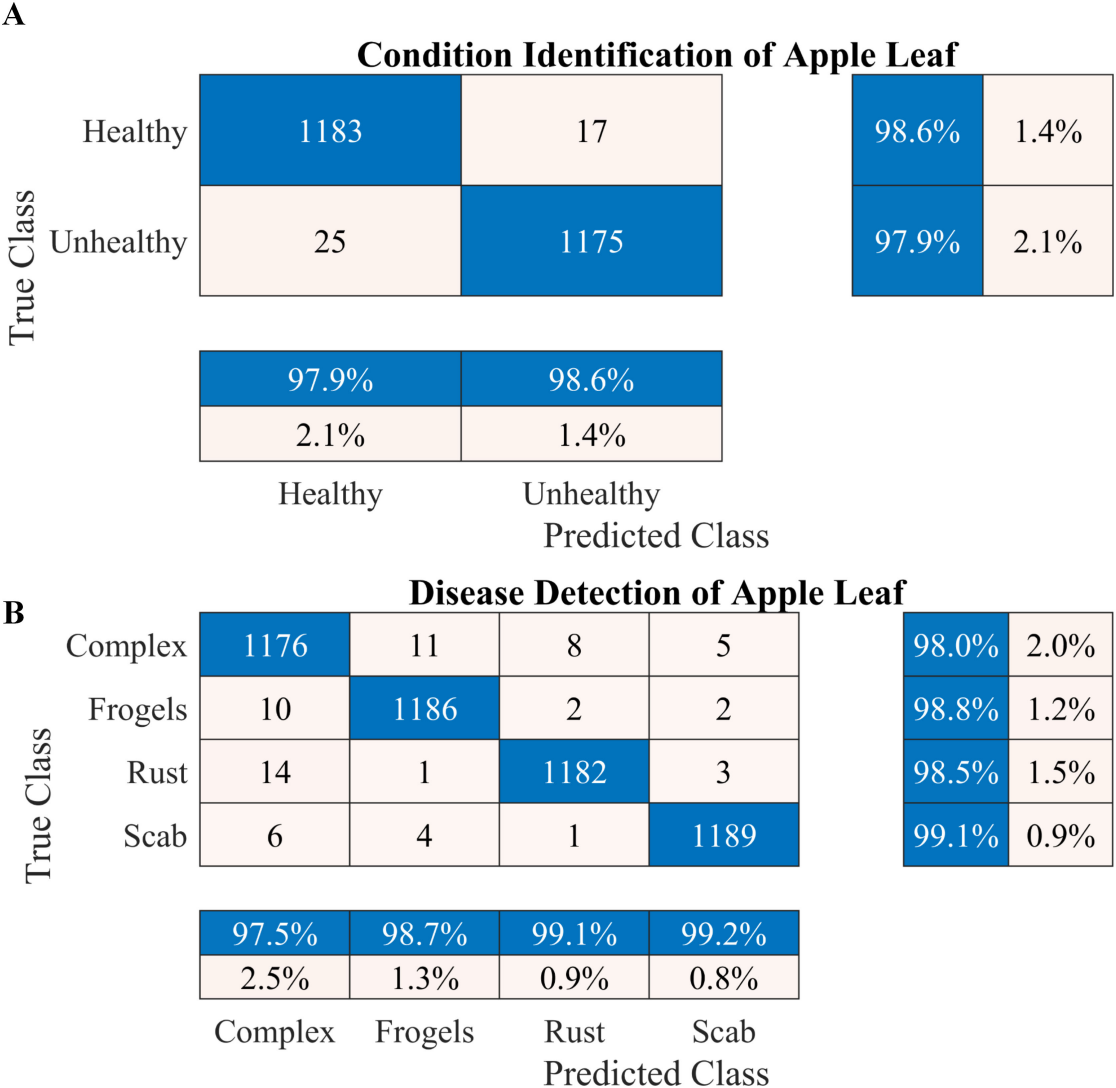
Performance of the proposed two-stage methodology. **(A)** Apple leaf condition identification and **(B)** apple leaf disease detection.

**Figure 5:**
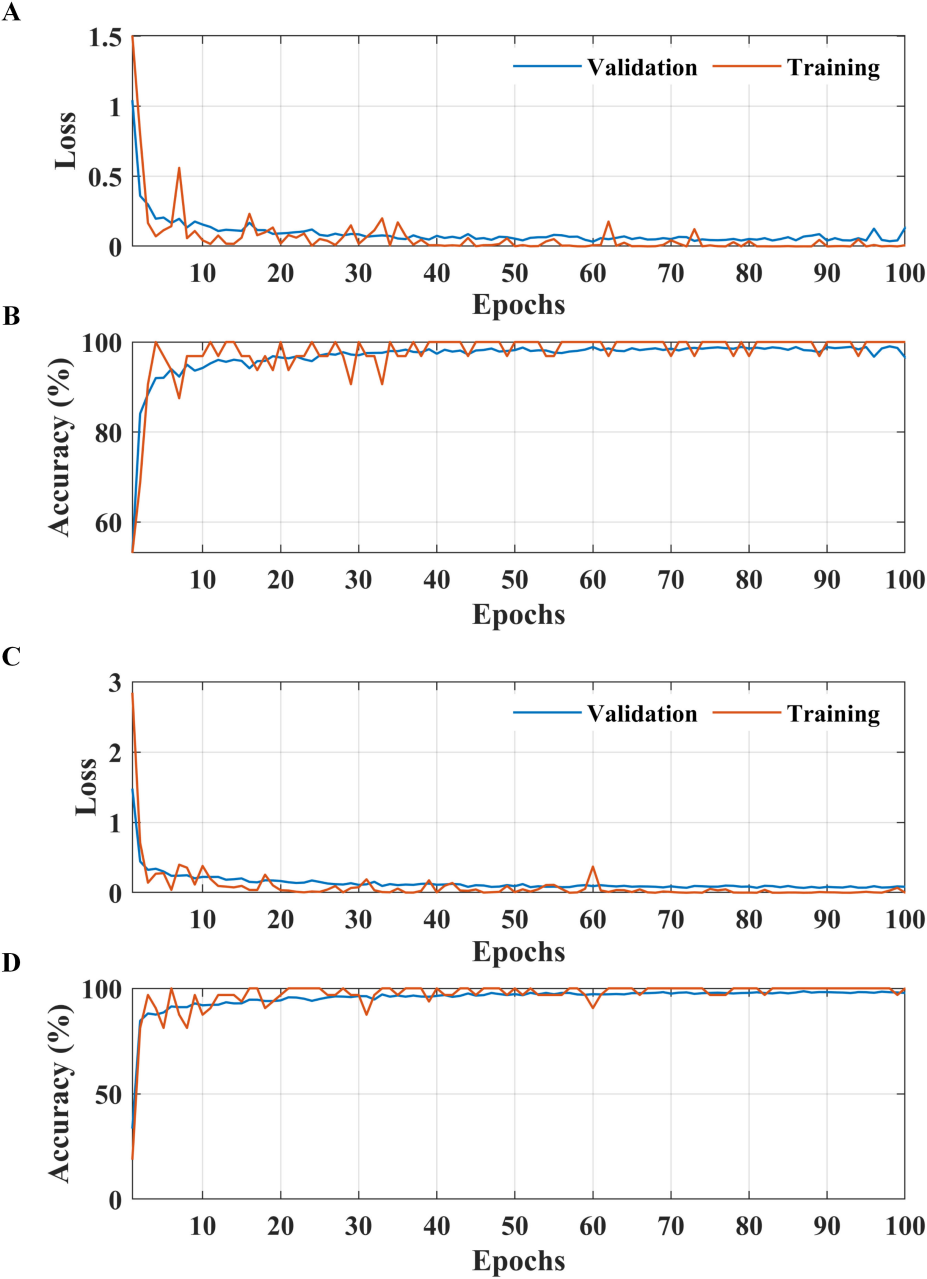
Learning curves of the proposed two-stage methodology. **(A, B)** Apple leaf condition identification model. **(C, D)** apple leaf disease detection model.

The data presented in **Table 4** indicate that the DenseNet-20 produced the highest classification accuracy of 94%, with a training time of approximately 45 h. Conversely, the proposed model yielded a reasonable classification rate of 91.02%, with only 1 h of training time. The proposed model exhibited an acceptable precision rate (**Table 2**) and accurately classified 875 of the 925 healthy apple leaf images. Furthermore, the proposed model used the least number of learnable parameters (1.3 million) in comparison with other models. Therefore, the proposed framework was used to further validate the identification and disease detection rate, as discussed in Section 2.1. After identifying the condition of an apple leaf (healthy or diseased), the original type of disease was detected using the proposed framework. The data were augmented to balance the dataset (**Table 2**); **Figures 4** and **5** illustrate the corresponding results.

**Figure 6:**
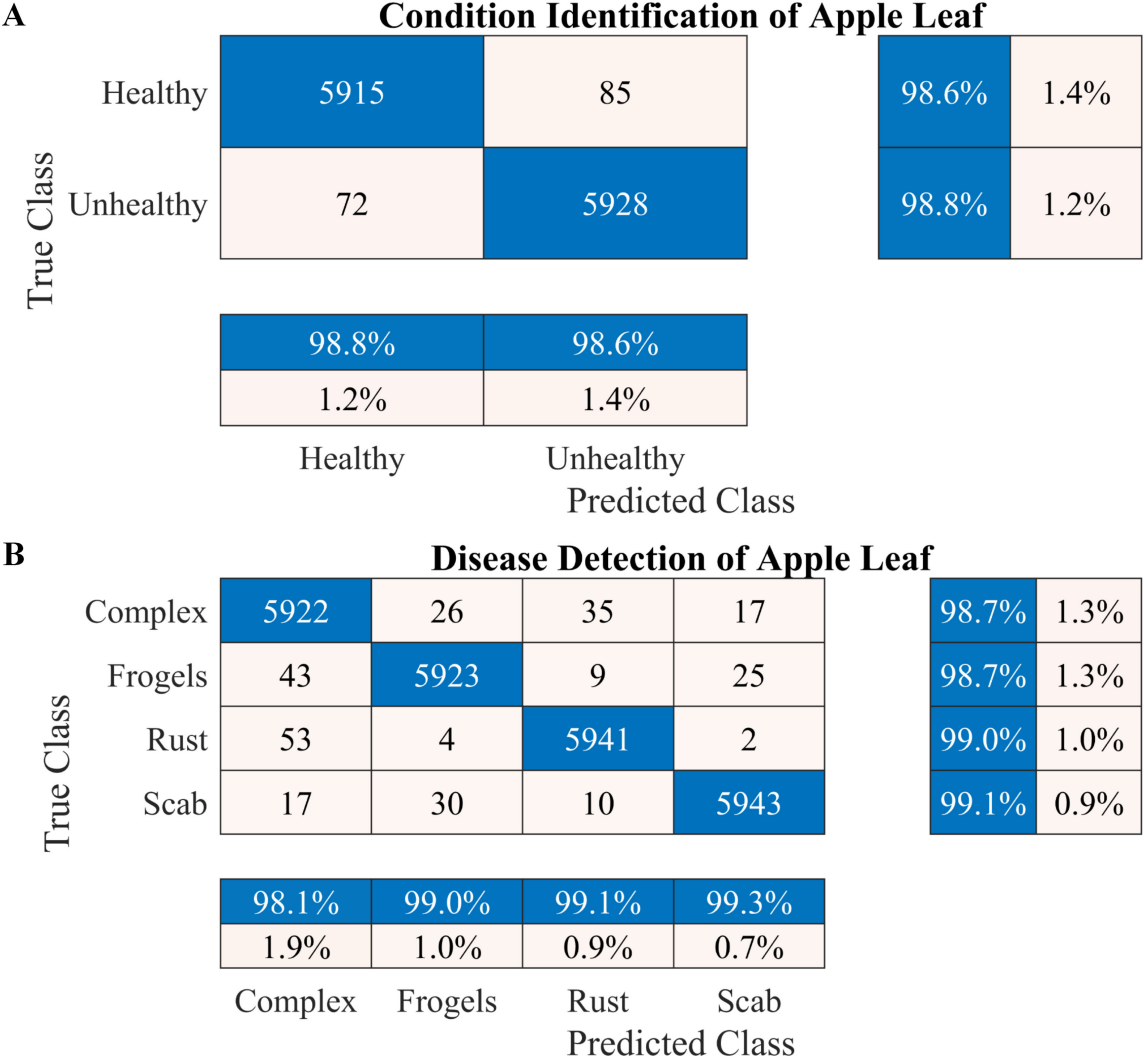
Performance of the proposed two-stage methodology based on the 10-fold cross-validation. **(A)** Apple leaf condition identification and **(B)** apple leaf disease detection.

According to the results presented in **Figure 4A**, the proposed methodology increased the condition identification rate of apple leaves. Only 17 of 1200 leaves were misclassified in the healthy class, and 1175 of 1200 leaves were accurately classified in the diseased class, increasing the accuracy to 98.25%. Furthermore, in the detection of complex diseases in leaves (**Figure 4B**), 24 false negatives and 30 false positives suggested misclassification to some extent; however, the error was relatively low compared with true positives. In the case of frogeye leaf spots, 14 false positives and 4 false negatives indicated adequate performance with minimal misclassification. In the case of scab detection, 11 false positives and 7 false negatives suggested high accuracy. **Figure 5** depicts the learning curves of both trained models, which stabilized after approximately 40 epochs. **Figure 6** shows the 10-fold cross-validation results, demonstrating the effectiveness of the proposed two-stage approach against overfitting. A comparison of the proposed approach with those reported in the literature is presented in **Table 5**.

**Table 5:** Comparison of the proposed framework with those reported in other studies using the Plant Pathology 2021 - FGVC8 dataset.

Study	Results (%)
(Yadav et al., 2022)	92.66
(Yu et al., 2022)	95.7
(Feng et al., 2023)	90.49
(Ullah et al., 2024)	96.4
(Ait Nasser and Akhloufi, 2024)	95.96
(Li et al., 2024)	95.69
Proposed	98.25% (for apple leaf condition identification) 98.60% (for apple leaf disease detection)

## 4 Discussion

The global agricultural industry plays a vital role in ensuring food security, and the detection of plant diseases is crucial for maintaining crop productivity and sustainability (Feng et al., 2023; Li et al., 2024). The accurate identification of leaf diseases in apple trees is critical for timely intervention and yield optimization. This paper presents a novel lightweight deep learning model and framework designed to efficiently recognize and classify diseases in apple leaves, offering a valuable tool for agricultural stakeholders.

We used a lightweight deep learning model and framework for apple leaf condition identification and disease detection. An ablation study (**Table 3**) was performed to determine the optimal layer configuration, which indicated that increasing or decreasing the number of layers affected the training time and classification accuracy. Notably, the 37-layer deep learning architecture achieved the highest validation accuracy (91.02%) with reduced validation loss for the original dataset (without augmentation). Furthermore, the training time of the proposed model was significantly shorter than that of the other deep learning models (**Table 4**) despite using only 1.3 million learnable parameters. Moreover, the proposed model was substantially lighter than others. Despite the increased efficiency and lightweight characteristics, the proposed model maintained competitive performance in terms of precision, recall, and accuracy. This reduced memory and storage requirements, rendering it suitable for deployment in devices with limited resources. This balance between efficiency and performance can be crucial for practical applications.

To increase the accuracy, we proposed a two-stage architecture using the selected lightweight 37-layer deep learning model. The primary idea was to validate the hypothesis that transfer learning benefits from correlated images using frozen CNN weights. In the first stage, the proposed lightweight model was designed and trained to identify the condition of the apple leaf (healthy or diseased). The model performed well in classifying healthy and diseased apple leaves, with a classification accuracy of 98.6% (**Figure 4A**). In the second stage, the model trained in Stage I was reused using its frozen weight (transfer learning concept) for diseased leaf subclassification into rust, complex, scab, and frogeye leaf spots. The use of frozen weights on correlated images facilitated the subclassification process. As indicated in **Figure 4B**, the performance of the diseased leaf subclassification significantly increased to 99.2%, with the true positive rate exceeding 98% for all subclasses. A comprehensive 10-fold cross-validation analysis was performed to further assess the robustness of the model against data leakage and overfitting. The results demonstrated a consistently high classification accuracy (**Figure 6**), further confirming the efficacy of the proposed two-stage framework.

The comparative analysis further revealed that the proposed model and framework yield a better classification performance than the other models utilizing the same dataset (**Table 5**). The proposed model offers a practical solution for plant disease classification by balancing performance, efficiency, and resource requirements. This makes it a valuable tool for real-world applications, where rapid and accurate plant disease identification and detection are essential.

Certain limitations were observed in this study. We focused solely on image data to classify apple leaf conditions and diseases. However, in the future, image data should be combined with spectral or genomic data to further enhance classification and robustness. Furthermore, existing architectures employed for plant disease classification are relatively simple. Exploring more advanced architectures, such as CNNs with attention mechanisms or transformer-based models, may improve performance. Another key limitation of this study was the validation of the proposed framework using a single dataset. Future studies should prioritize testing the generalizability of the framework across diverse datasets by incorporating various environmental and confounding factors to ensure broader applicability.

## 5 Conclusions

This study proposes a lightweight deep learning model and framework for identifying the apple leaf condition (healthy or diseased) and detecting diseases (e.g., rust, complex, scab, and frogeye leaf spots). A 37-layer lightweight deep learning model was designed to identify the apple leaf conditions, and the Plant Pathology 2021 - FGVC8 dataset available online was used for validation. Image augmentation techniques were used to balance the classes. The proposed model was trained using an augmented dataset, and numerous comparative experiments were performed considering various performance evaluation indicators. The experimental results demonstrated that the proposed method achieved a high accuracy of 98.25% for identifying the apple leaf condition. Furthermore, the proposed lightweight deep learning model required considerably fewer learnable parameters than other models. The trained model was reused to evaluate its performance in disease class subclassification using transfer learning. The model achieved a high classification accuracy of 98.60% for actual disease detection. This excellent classification performance confirmed that the proposed model outperformed existing deep learning algorithms, providing superior results in apple leaf disease detection tasks. The study findings serve as a reference for classifying agricultural diseases using deep learning techniques as the developed model is lightweight, rapid, and resilient.

## Data Availability

The original contributions presented in the study are included in the article/supplementary material. Further inquiries can be directed to the corresponding author.
